# Wound infiltration with ropivacaine as an adjuvant to patient controlled analgesia for transforaminal lumbar interbody fusion: a retrospective study

**DOI:** 10.1186/s12871-020-01205-5

**Published:** 2020-11-18

**Authors:** Kunpeng Li, Changbin Ji, Dawei Luo, Hongyong Feng, Keshi Yang, Hui Xu

**Affiliations:** Department of Orthopaedics, Liaocheng People’s Hospital, No 67 Dongchang West Road, Liaocheng City, 252000 Shandong Province China

**Keywords:** Wound infiltration, Ropivacaine, Pain management, PCA, Transforaminal lumbar interbody fusion

## Abstract

**Background:**

Surgical procedure usually causes serious postoperative pain and poor postoperative pain management negatively affects quality of life, function and recovery time. We aimed to investigate the role of wound infiltration with ropivacaine as an adjuvant to patient controlled analgesia (PCA) in postoperative pain control for patients undergoing transforaminal lumbar interbody fusion.

**Methods:**

One hundred twelve patients undergoing lumbar fusion were retrospectively reviewed and divided into two groups (ropivacaine and control groups) according to whether received wound infiltration with ropivacaine or not. Visual Analogue Scale (VAS) score, analgesics consumption, number of patients requiring rescue analgesic, hospital duration and incidence of complications were recorded. Surgical trauma was assessed using operation time, intraoperative blood loss and incision length.

**Results:**

The amount of sufentanil consumption in ropivacaine group at 4 h postoperatively was lower than that of control group (24.5 ± 6.0 μg vs 32.1 ± 7.0 μg, *P* < 0.001) and similar results were observed at 8, 12, 24, 48 and 72 h postoperatively(*P* < 0.001). Fewer patients required rescue analgesia within 4 to 8 h postoperatively in ropivacaine group (10/60 vs 19/52, *P* = 0.017). Length of postoperative hospital durations were shorter in patients receiving ropivacaine infiltration compared to control cohorts (6.9 ± 0.9 days vs 7.4 ± 0.9 days, *P* = 0.015). The incidence of PONV in ropivacaine group was lower than that in control group (40.4% vs 18.3%, *P* = 0.01). However, VAS scores were similar in two groups at each follow-up points postoperatively, and no difference was observed(*P* > 0.05).

**Conclusion:**

Wound infiltration with ropivacaine effectively reduces postoperative opioid consumption and PONV and may be a useful adjuvant to PCA to improve recovery for patients undergoing lumbar spine surgery.

## Background

Transforaminal lumbar interbody fusion (TLIF) has been widely used in treatment of lumbar degenerative spine disorders, and achieved good clinical results and high patient satisfaction [1, 2]. It can decompress the nerve roots, immobilize the instrumented segments and provide stability of spine. Despite its good outcome, surgical procedure usually causes serious postoperative pain and poor pain management negatively affects quality of life, function, and recovery [3].

Traditionally, patient-controlled analgesia (PCA) has been identified as effective in postoperative pain management and is the most frequently used analgesic method for spine surgery [4, 5]. However, the main drug used is opioid analgesics, which have severe side effects including nausea or vomiting, confusion, urinary retention, sedation, respiratory depression, and pruritus [6]. Therefore, finding other analgesic strategies with fewer potentially adverse effects will be beneficial for patients suffering from postoperative pain.

In recent years, wound infiltration with local anesthetics has become an attractive method in postoperative analgesia because of its safety, simplicity and low-cost [7, 8]. As a local anesthetic, ropivacaine is a propyl analog of bupivacaine with longer duration of action and much safer cardiotoxicity profile. Several reports [9, 10] have confirmed that wound infiltration with ropivacaine could significantly reduce postoperative pain, mitigate supplemental analgesic demand as well as curtail hospital stay following some surgeries, such as joint replacement, abdominal surgeries, and cesarean deliveries.

With the ideal analgesic modality for lumbar fusion surgery still unknown and possibly simpler alternatives to local infiltration analgesia gaining popularity, it is imperative to clarify the role of wound infiltration with ropivacaine in managing postoperative pain after this procedure. We hypothesized that wound infiltration with ropivacaine as an adjuvant to PCA for patients undergoing TLIF is more effective than PCA alone, resulting in lower postoperative pain scores, less consumption of opioid medications and lower incidence of postoperative nausea and vomiting (PONV) compared with PCA alone.

## Methods

### Patient population

This retrospective cohort study was conducted in Liaocheng People’s Hospital. All patients were identified to undergo single-level TLIF procedure with a single surgeon between January 2016 and December 2018. The inclusion criteria were as follow: age 18–65 years, primary diagnosis of lumbar disc herniation, lumbar spinal stenosis and lumbar degenerative spondylolisthesis (grade 1), single level TLIF (L3/4, L4/5 or L5/S1), American Society of Anesthesiologists (ASA) grade I to II. Patients were excluded according to the exclusion criteria: allergic to ropivacaine, preoperative opioid consumption in last 3 months, and history of spine surgery.

Enrolled patients were divided into two groups, ropivacaine group and control group. Each patient provided written informed consent before enrollment. The study was approved by the Ethics Committee of Liaocheng People’s Hospital.

### Surgical procedure

The procedure was performed as described by Ge [11]. All procedures were carried out under controlled general anesthesia with endotracheal intubation. Each patient was positioned prone on a radiolucent operating table after induction of general anesthesia. Radiographs were used to check the operation level. A midline approach was used to expose the lamina and spinous processes. Bilateral pedicle screws were placed and a rod was sited using special persuaders. The laminectomy and facetectomy were then performed at the level. The cartilaginous material was removed from the endplates using the scraper. The autogenous morselized bone from the laminae and processus articularis was placed into the anterior intervertebral space. This was followed by the implantation of cage plus autogenous bone. The wound was copiously irrigated and closed in layers. Routine monitoring included electrocardiography, pulse oximetry, blood pressure, and arterial blood gas analysis. All patients received general anesthesia with 0.1% propofol, dexmethetomedine, fentanyl, remifentanil and cisatracurium. No preemptive scheduled analgesic regimen was employed.

### Wound infiltration with ropivacaine

Just before closure, 10 ml ropivacaine (concentration: 0.75%) was infiltrated in paravertebral muscles, subcutaneous, and cutaneous tissue along each side of the wound edges. At the end of surgery, patients were turned to supine position, and extubated successfully on the table. Once awake and responded to verbal commands, patients were transferred to the post-anesthesia care unit (PACU). After PACU, patients were transferred to spine ward for further monitoring and recovery care.

### Postoperative management

All patients received intravenous PCA with 0.8 μg/ml of sufentanil for 72 h. The sufentanil was administrated via PCA pump at a bolus of 2 ml (1.6μg) with a 5 min lockout time and the maximum dosage was 12.8μg per hour. Flurbiprofen axetil was injected as a rescue analgesic when requested by patients with visual analogue scale (VAS) scores≥5.

Patients were routinely administered prophylactic antibiotics for 24 h and encouraged to start out-of-bed activities with braces within 3 days after surgery. Mechanical thomboprophylaxis was given to prevent phlebothrombosis of both legs. Since discharge from the hospital, all patients were clinically and radiologically assessed in outpatient clinic every 3 months.

### Observation index

Primary outcome was total sufentanil consumption over the first 72 h. The sufentanil consumption can be calculated by multiplying the volume by the concentration (0.8μg/ml, total 200μg sufentanil in 250 ml saline). The volume of saline was shown in the PCA pump. Secondary outcome measures were VAS scores, number of patients requiring flurbiprofen axetil as analgesic rescue and incidence of complications including PONV and wound infection. Operation time, intraoperative blood loss, incision length and length of postoperative hospital duration were also recorded in the data.

### Sample size and statistical analyses

The sample size was determined for the primary outcome measure. According to previous study [12], a difference of more than10ug in sufentanil consumption at 24 h after surgery between groups was considered clinically relevant. Under the assumption that the standard deviation is 16μg, a sample size of 41 per group was determined (power = 80%, *p* = 0.05). For the secondary outcome of VAS score, sample size estimates were also considered. On the basis of the data from clinical practice and the study by Elder et al. [13], the standard deviation for the VAS was assumed to be 2.0 and a sample size of 38 patients per group would provide statistical power of 80% to detect a difference between groups of 1.3. Additionally, we reviewed several similar reports [14, 15] and found that the number of treated subjects was approximately 40 to 50. Therefore, we determined that a total of 50 patients per group were enrolled. The power was 0.872 according to primary outcome data mentioned above (*n* = 50, σ = 16, δ = 10, α = 0.05). The sample size and power analysis were performed using Power and Sample Size Calculation version 3.1.6.

The SPSS 22.0 statistical package (SPSS, Chicago, IL, U.S.A.) was used for statistical analyses. Continuous data were presented as the mean ± standard deviation and analyzed using two-sample t test and ANOVA analysis. Chi square test was performed to analyze count data. For all analyses, a *P* value < 0.05 was considered statistically significant.

## Results

This clinical trial enrolled 112 patients undergoing single-level TLIF procedure, 60 patients who received wound infiltration with ropivacaine in ropivacaine group and 52 without ropivacaine infiltration in control group. Patients’ demographics and basic characteristics, including age, gender, weight, height, body mass index (BMI), primary diagnosis and operation level were shown in the Table 1, and no significant difference was observed between two groups.

**Table 1:** Characteristics of the patient cohort in two groups

Parameter	Ropivacaine group	Control group	*P* value
Number of patients	60	52	
Age (years)	57.9 ± 7.7 (41–73)	56.9 ± 7.5 (42–72)	0.529
Gender, males/females	39/21	35/17	0.799
Weight (kg)	67.2 ± 8.2	66.3 ± 8.9	0.581
Height (cm)	167.9 ± 6.5	168.8 ± 6.5	0.472
BMI (kg/m^2^)	23.8 ± 2.0	23.2 ± 2.1	0.134
Diagnosis			0.775
LDH	26	32	
LSS	17	16	
LDS	9	12	
Operation level			0.790
L3/4	6	5	
L4/5	25	32	
L5/S1	21	23	
Operation time (min)	103.1 ± 15.2	106.5 ± 17.1	0.260
Intraoperative blood loss (ml)	250.6 ± 38.6	258.1 ± 39.7	0.314
Incision length (cm)	6.2 ± 0.7	6.1 ± 0.8	0.299
Postoperative hospital duration (day)	6.9 ± 0.9	7.4 ± 0.9	0.015

*LDH* Lumbar disc herniation, *LSS* Lumbar spinal stenosis, *LDS* Lumbar degenerative spondylolisthesis

### Operation index and hospital duration

The mean operation time was 103.1 ± 15.2 min and 106.5 ± 17.1 min in ropivacaine and control groups respectively, and no difference was found (*P* = 0.26). There was also no difference in incision length and intraoperative blood loss between two groups (*P* = 0.299 and *P* = 0.314) (Table 1).

For patients receiving ropivacaine infiltration, length of postoperative hospital duration was 6.9 ± 0.9 days, which was shorter than that of control cohort (7.4 ± 0.9 days), and significant difference was detected between two groups (*P* = 0.015) (Table 1).

### Postoperative analgesics consumption

The amount of sufentanil consumption at 4 h postoperatively in ropivacaine group was lower than that in control group (*P* < 0.001). Significant difference was also found in the cumulative sufentanil consumption between two groups at 8, 12, 24, 48 and 72 h after surgery (*P* < 0.001).

**Fig. 1:**
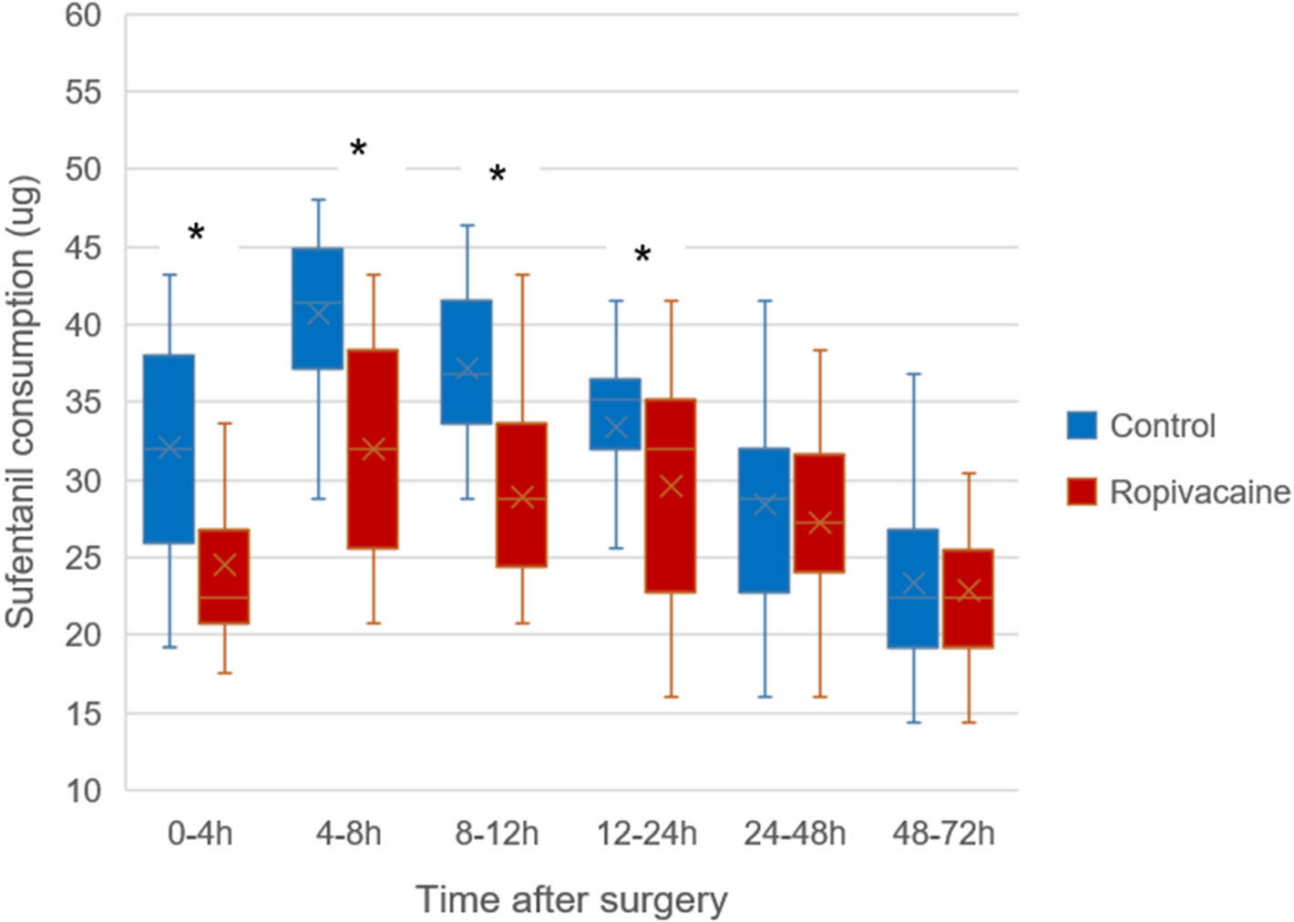
Boxplot showing sufentanil consumption in the postoperative period for two groups. The boxes indicate the interquartile range, the crosses within the boxes indicate the median, and the whiskers indicate the range. The asterisks indicate significance(*P* < 0.05). h = hours postoperatively

Patients in the ropivacaine group consumed less sufentanil than those in the control group within 4 to 8 h postoperatively (*P* < 0.001). Similar results were observed within 8 to12 hours and 12 to 24 h postoperatively (*P* < 0.001 and *P* = 0.001). However, no difference was detected within 24 to 48 h and 48 to 72 h postoperatively (*P* = 0.276 and *P* = 0.547) (Fig. 1).

There were five patients who required flurbiprofen axetil as rescue in the ropivacaine group and eight in the control group within first 4 h postoperatively, and no difference was found(*P* = 0.245). However, fewer patients in ropivacaine group needed analgesic rescue within 4 to 8 h postoperatively compared to control group (*P* = 0.017). Within 8 to 12 h postoperatively, no difference was found between two groups (*P* = 0.101) (Table 2).

**Table 2:** The administration of flurbiprofen axetil in two groups

Period	Ropivacaine group	Control group	*P* value
First 4 h	5/60	8/52	0.245
From 4 to 8 h	10/60	19/52	0.017
From 8 to 12 h	6/60	11/52	0.101

### Pain evaluation

**Fig. 2:**
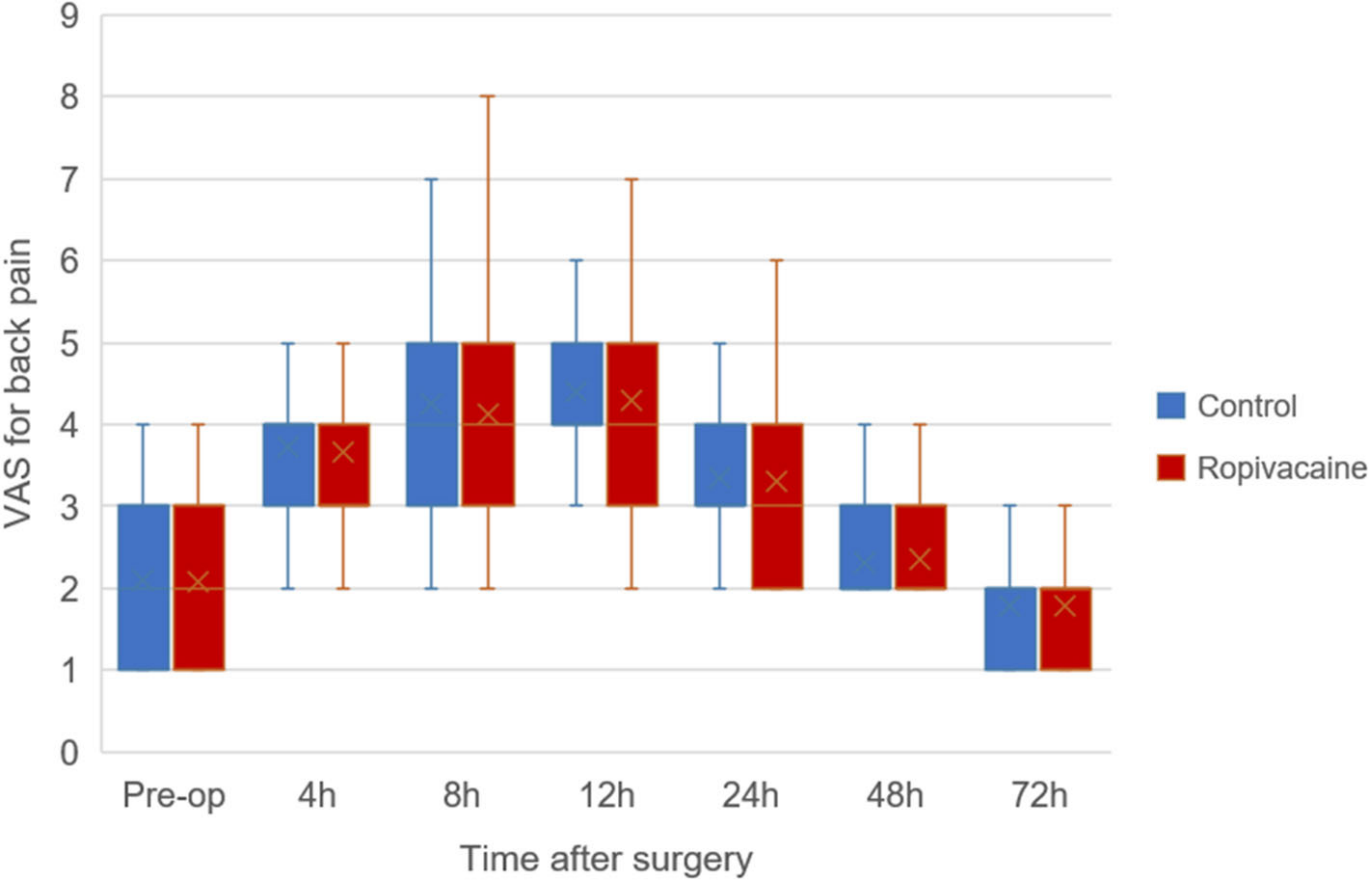
Boxplot showing VAS scores for back pain over the first 72 h postoperatively for two groups. The boxes indicate the interquartile range, the crosses within the boxes indicate the median, and the whiskers indicate the range. No significant difference was observed at each time between two groups(*P* > 0.05). h = hours postoperatively

Average VAS scores at 4 h postoperatively was 3.7 ± 1.5 points in ropivacaine group and 3.7 ± 1.3 points in control group, and no difference was observed (*P* = 0.808). Similar results were shown between the ropivacaine and control groups at 8 h (4.1 ± 1.3 vs 4.3 ± 1.1, *P* = 0.568), 12 h (4.3 ± 1.3 vs 4.4 ± 1.2, *P* = 0.655), 24 h (3.3 ± 1.1 vs 3.4 ± 1.0, *P* = 0.822), 48 h (2.4 ± 0.6 vs 2.3 ± 0.5, *P* = 0.700) and 72 h (1.8 ± 0.6 vs 1.8 ± 0.6, *P* = 0.964) postoperatively (Fig. 2).

### Complications

The incidence of PONV in the ropivacaine group was lower than that in the control group (18.3% vs 40.4%, *P* = 0.010). Only one patient had wound infection in each group and no difference was detected (*P* = 0.919, Table 3). These two patients recovered after routine antibiotic treatment and dressing change. No clinical deterioration, permanent morbidity or mortality occurred in this study. There was also no hardware failure, nerve root injury, cerebrospinal fluid leakage, and adjacent segment disc herniation over the follow-up.

**Table 3:** The incidence of complications in two groups

Complication	Ropivacaine group	Control group	*P* value
PONV	11/60	21/52	0.010
wound infection	1/60	1/52	0.919

## Discussion

In this study, we adopted wound infiltration with ropivacaine as an adjuvant to PCA in TLIF procedure to assess its efficacy in postoperative pain management. Results showed better outcomes in patients who received the wound infiltration with ropivacaine. It reduced the consumption of opioid drugs via PCA after TLIF procedure and decreased the number of patients who required rescue analgesia, while achieving similar pain relief. The data further indicated a lower incidence of PONV and a shorter hospital duration in the ropivacaine group.

Currently, PCA is the most widely used approach to manage postoperative pain after spine surgery. However, the use of opioids in PCA is often associated with adverse effects. So multimodal pain management is recommended in order to reduce opioid-related adverse effects. Since Mullen and Cook first demonstrated the use of wound infiltration with local anesthetics in spine surgery in 1979 [16], a few literatures reported the application of wound infiltration with local anesthetics in several surgical procedures. Koehler [15] performed a randomized controlled trial and reported that surgical-site injection with a multimodal cocktail could reduce narcotic utilization and provide improved pain control, with no adverse effects attributable to the local injection. Similarly, in a retrospective study of patients undergoing thoracolumbar junction fracture surgery, Swennen [17] found that local infiltration analgesia had a reduction of VAS and morphine consumption in postoperative pain control. Another two studies [13, 18] on the continuous analgesic infusion also demonstrated better outcomes in managing postoperative pain with less opioid use and lower pain scores.

The main outcome measures in this study were visual analogue pain score and opioid usage. In the current study, results showed patients receiving ropivacaine infiltration reported similar VAS scores and less sufentanil consumption via PCA at each follow-up point within 72 h after surgery, which was not completely consistent with previous reports. Although similar VAS score was reported in two groups, less opioid use was another important indicator that reflected the decreased postoperative pain in ropivacaine group. We further found that periodic consumption of sufentanil in the ropivacaine patients was less than that of the control group only within 24 h postoperatively. We attributed this to that the single dose of ropivacaine was injected in our study and its duration acted within 24 h [19]. This also indicated that the decreased opioid consumption would be associated with the use of wound infiltration with ropivacaine.

There are multiple choices for local anesthetic. Bupivacaine and ropivacaine are commonly used after surgery, but ropivacaine is reported to have a lower risk of cardiovascular or central nervous system toxicity [20]. Elder [18] used an elastomeric pump to infuse 0.5% bupivacaine into the wound for pain control in lumbar spinal fusion and data showed that continuous bupivacaine infusion resulted in lower pain scores and narcotic use with lower incidence of nausea and vomiting and decreased times to mobility and functional independence. He also considered that a single intraoperative dose of local anesthetic does not provide adequate postoperative pain control because of the short period of analgesic effect inherent to local anesthetics. But Sun’s study reported that local wound infiltration with single dose of ropivacaine after open hepatectomy could improve postoperative pain relief, reduce surgical stress response, and accelerate postoperative recovery. Our study confirmed that intraoperative wound infiltration with single dose of ropivacaine could provide pain relief and reduce opioid use within postoperative 24 h. However, a small number of studies have demonstrated contrary results showing wound infiltration with ropivacaine does not offer significant postoperative pain relief [21, 22]. Kakagia [23] compared local infiltration of ropivacaine with levobupivacaine in a randomized controlled trial and found that in terms of intensity and duration of analgesia, ropivacaine was less effective than levobupivacaine in reducing postoperative pain associated with mini abdominoplasty.

PONV is a common side effect of opioid-based intravenous PCA. Previous studies reported that a logarithmic dose response relationship between the use of postoperative opioids and PONV [24, 25]. The decreased rate of PONV that was observed in ropivacaine group may be related to less opioid consumption consumed by patients receiving wound infiltration with ropivacaine, which was similar to Li’s report from a randomized controlled trial [26]. PONV was also an unpleasant side effect feared by many patients during acute postoperative course, which can also cause dehydration, electrolyte imbalance, postoperative bleeding, wound dehiscence, and pulmonary aspiration [27, 28]. Hence, in addition to improving patient experience, the lower rate of PONV may also contribute to shorter length of hospital duration.

Effective pain management is now recognized as one of the three fundamental aspects of enhanced recovery after surgery [29]. In this trial, we investigated the role of wound infiltration with ropivacaine in postoperative hospital duration, which was reported to be a better indicator of patient recovery [30]. Patients who received wound infiltration with ropivacaine as an adjuvant to PCA had shorter length of postoperative hospital duration compared to those receiving PCA alone. These suggested that the use of ropivacaine infiltration may promote enhanced recovery and further decrease postoperative hospital stay in patients undergoing TLIF, which may be attributed to the decreased opioid use of PCA after wound infiltration with ropivacaine.

There are some limitations to this study, which may impair the ability to assess the effect of wound infiltration with ropivacaine on postoperative pain management. First, this was a retrospective cohort study in a single center, not randomized and blind, which may introduce the possibility of selection bias. Second, we did not analyze the difference of time to administrate rescue analgesic. Moreover, this study enrolled a small patient population. In the future, prospectively randomized controlled study, including more patients in multi-center will be performed to properly evaluate the role of wound infiltration with ropivacaine as an adjuvant to PCA in postoperative pain management.

## Conclusion

Results of this study indicate that wound infiltration with ropivacaine effectively reduces postoperative opioid consumption and PONV and may be a useful adjuvant to PCA to improve recovery for patients undergoing lumbar spine surgery.

## Data Availability

All relevant data was presented within the manuscript and the datasets used and/or analyzed during the current study are available from the corresponding author on reasonable request.

## Abbreviations

TLIF: Transforaminal lumbar interbody fusion
PCA: Patient-controlled analgesia
ASA: American Society of Anesthesiologists
PACU: Post-anesthesia care unit
VAS: Visual analogue scale
PONV: Postoperative nausea and vomiting
